# Therapeutic potential of 4-hexylresorcinol in reducing sarcopenia in diabetic masseter muscle

**DOI:** 10.1186/s40902-025-00457-w

**Published:** 2025-01-20

**Authors:** Dhouha Gaida, Young-Wook Park, Yei-Jin Kang, Seong-Gon Kim

**Affiliations:** Gangneung–Wonju National University KR, Gangneung-si, Gangwon-do Republic of Korea

**Keywords:** Diabetes, Sarcopenia, 4-Hexylresorcinol, Masseter muscle, AMPK, Glut4

## Abstract

**Background:**

This study aimed to evaluate the effects of 4-hexylresorcinol (4HR), a synthetic compound with antioxidant and stress-modulating properties, on diabetic sarcopenia in the masseter muscle.

**Methods:**

A controlled, parallel-arm study was conducted using 38 Sprague–Dawley rats divided into diabetic and non-diabetic groups. Diabetes was induced with streptozotocin (STZ), and the groups were further subdivided to receive weekly subcutaneous injections of either 4HR or saline. Muscle volume was assessed using micro-computed tomography (μCT), and glycogen storage and protein expression were analyzed using periodic acid-Schiff (PAS) staining and immunohistochemistry.

**Results:**

μCT analysis revealed that diabetic rats exhibited significantly reduced masseter muscle volume compared to non-diabetic rats. However, 4HR treatment partially mitigated muscle volume loss in diabetic animals. Histological analysis showed higher PAS staining intensity in the diabetic group treated with 4HR compared to the untreated diabetic group, suggesting improved glycogen storage. Immunohistochemistry demonstrated that 4HR treatment significantly increased Glut4 and phosphorylated AMPKα (p-AMPKα) expression in diabetic muscle, indicating enhanced glucose uptake and metabolic activity.

**Conclusions:**

4HR effectively alleviates diabetes-induced sarcopenia by preserving muscle volume, enhancing glycogen storage, and upregulating Glut4 and p-AMPKα expression. These findings suggest that 4HR holds potential as a therapeutic agent for combating muscle wasting in diabetes.

## Background

Sarcopenia, defined as the loss of muscle mass and function, is frequently observed as part of the aging process [1]. It is associated with reduced mobility, increased risk of falls, and decreased quality of life in elderly individuals. The overall prevalence of sarcopenia has been reported to range from 10 to 40% [2]. Interestingly, its prevalence is two to three times higher in diabetic patients [3, 4], indicating a strong link between diabetes and muscle degeneration. In diabetic patients, decreased insulin secretion or insulin resistance has been considered an underlying cause of sarcopenia [5].

Various cellular mechanisms have been proposed to explain diabetic sarcopenia [5]. Understanding these mechanisms is crucial for developing effective therapeutic strategies. Increased blood glucose levels may activate Krüppel-like factor 15, leading to increased apoptosis of muscle cells [6]. In diabetes, the nuclear factor kappa B (NF-κB) signaling pathway is activated [7], and the expression level of tumor necrosis factor-α (TNF-α) is increased [8], both of which contribute to muscle inflammation and degradation. Additionally, decreased expression of certain proteins in the transforming growth factor-β (TGF-β) superfamily, such as bone morphogenetic proteins (BMPs), has been associated with sarcopenia [9], suggesting that impaired muscle regeneration may play a role. Furthermore, diabetic patients exhibit suppression of the AMP-activated protein kinase (AMPK) signaling pathway and activating AMPK can improve these metabolic issues by enhancing insulin sensitivity, glucose transport, and lipid metabolism, making it a critical target for therapeutic interventions in diabetes [10, 11]. AMPK activation by natural compounds and pharmacological agents improves muscle cell proliferation and combats sarcopenia-related muscle loss [12]. Activation of the AMPK signaling pathway increases the acetylation level of histone 3, resulting in increased expression levels of Glut4, a glucose transporter essential for muscle glucose uptake [13].

4-Hexylresorcinol (4HR) is an organic compound derived from plants [14], known for its anti-inflammatory and potential therapeutic properties. 4HR is an inhibitor of the NF-κB signaling pathway [15], which is involved in the regulation of immune and inflammatory responses. Administration of 4HR inhibits the expression of TNF-α [16], thereby potentially reducing inflammation-induced muscle degradation. In addition, 4HR is a strong inducer of proteins in the TGF-β superfamily, such as TGF-β1 [17] and BMPs [18], which are important for muscle regeneration and repair. The administration of 4HR increases the expression levels of Glut4 and alleviates complications associated with diabetes [19], suggesting that it may enhance glucose uptake and improve muscle metabolism.

Given these properties, 4HR may have therapeutic potential in preventing or reversing diabetic sarcopenia. Hence, the present study examined the effect of 4HR on sarcopenia in the diabetic masseter muscle. The aim of this study was to evaluate the effect of 4HR administration on diabetic sarcopenia in the masseter muscle, a key muscle involved in mastication. First, muscle dimensions were compared using micro-computed tomography (μCT) to determine changes in muscle mass. Second, the expression levels of Glut4 and p-AMPKα were evaluated in the masseter muscle by immunohistochemistry to confirm in vivo effects. In addition, the intensity of periodic acid–Schiff (PAS) staining was also evaluated to assess glycogen storage in muscle tissue.

## Materials and methods

### Animal study

**Fig. 1:**
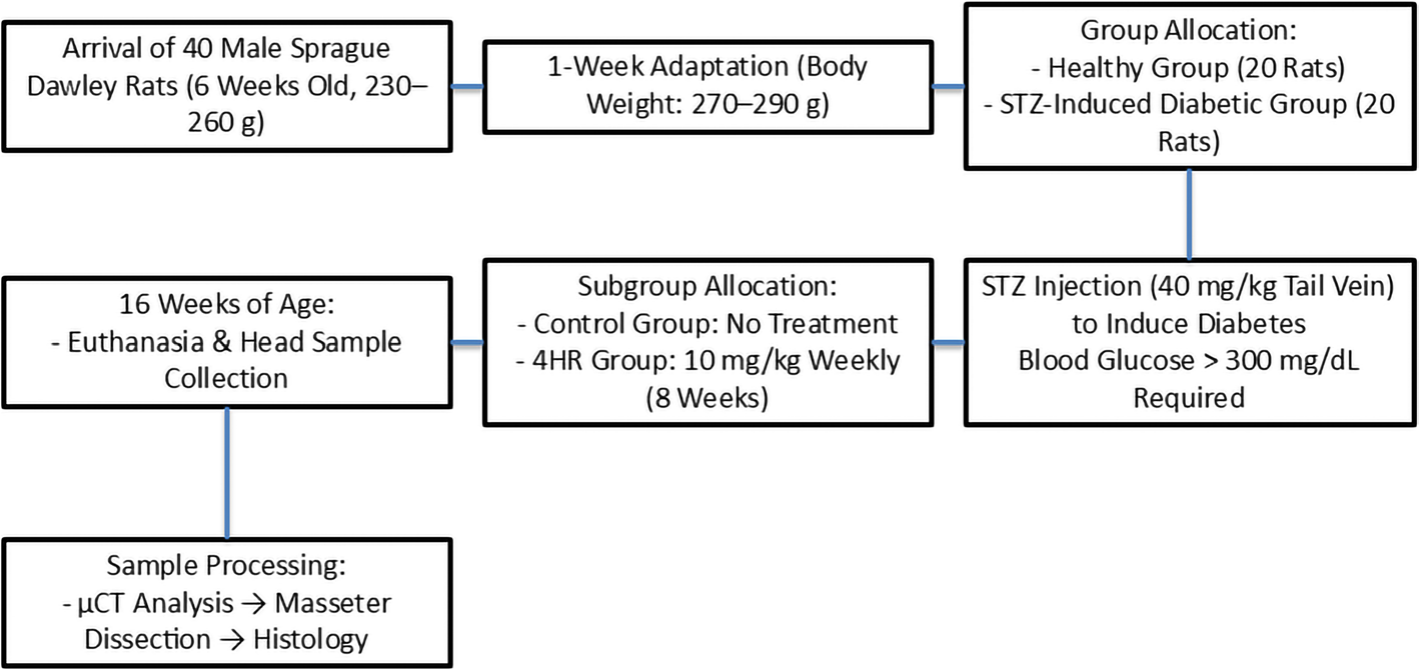
Schematic overview of experimental timeline

For this study, 38 head samples were obtained from a previous animal experiment (Fig. 1). All experimental procedures were conducted in compliance with the ethical guidelines of the Institutional Animal Care and Use Committee (IACUC) of Gangneung-Wonju National University, Gangneung, Republic of Korea. The study protocols were approved under approval numbers GWNU-2021–21 and GWNU-2021–22 on 23 November 2021. Initially, 40 male Sprague Dawley rats (seven weeks old, weighing 270–290 g) were included. However, two rats in the diabetic group died during the induction of diabetes, resulting in a final sample size of 38. The rats were divided into two main groups based on their health status: a healthy group (20 rats) and a diabetic group (18 rats). Type I diabetes was induced in the diabetic group through streptozotocin (STZ) injections. STZ was administered at a dosage of 40 mg/kg via tail vein injection. Three days after injection, fasting blood glucose levels were measured, and animals with glucose levels exceeding 300 mg/dL were included in the study. For cases where DM induction was unsuccessful, a repeated STZ injection was performed to ensure consistent induction. Only male rats were used in this study, as female rats are resistant to STZ-induced diabetes [20].

Each main group was further subdivided into two subgroups based on the treatment administered. One subgroup served as the control, while the other received 4HR treatment. In the healthy group, 10 rats were allocated to the control subgroup, and 10 rats received 4HR treatment. In the diabetic group, 9 rats were assigned to the control subgroup, and 9 rats received 4HR treatment. Thus, the final sample size for this study included 38 rats. The dosage of 4HR used in this study was carefully selected based on prior research and safety considerations. A weekly dose of 10 mg/kg was chosen, considering the LD_50_ values for rats and observed effects in other animals such as cats. Previous studies have demonstrated the efficacy of 4HR at dosages like this, showing significant effects like decreased blood sugar levels [20, 21]. Selecting a weekly dose of 10 mg/kg also facilitated practical dosage calculations and minimized potential errors, while avoiding complications that might arise from higher dosages due to the dose-dependent nature of the drug’s effects.

Samples were obtained from the heads of the euthanized male Sprague Dawley rats used in this study. Initially, the collected heads were subjected to μCT analysis to evaluate skeletal and muscular structures. Following μCT scanning, the masseter muscles were carefully dissected from the heads. The dissected muscle tissues underwent standard tissue processing procedures and were embedded in paraffin blocks. Serial sections of 5 µm thickness were prepared using a microtome for histological and immunohistochemical analyses.

### μCT scan and 3D analysis

Images were obtained using μCT50 (Scanco Medical, Brüttisellen, Switzerland) at the Center for Scientific Instruments, Gangneung-Wonju National University (Gangneung, Korea). For three-dimensional (3D) volume analysis, Digital Imaging and Communications in Medicine (DICOM) files obtained from the μCT scans of the 38 rats’ heads, were imported into ITK-SNAP software (version 4.0.2), an open-source tool designed for medical image segmentation. Manual segmentation of the masseter muscle was performed based on specific bony landmarks that define the anatomical boundaries of the muscle [22]. This process allowed for precise delineation of the muscle tissue from surrounding structures, facilitating accurate volume calculations. The use of consistent landmarks ensured that the measurements were reproducible and could be reliably compared between the control and experimental groups.

### PAS staining

To assess the glycogen content in the masseter muscle tissues, PAS staining was conducted using a PAS staining kit (Abcam, CAT#: ab150680). The tissue sections, which had been embedded in paraffin, were first deparaffinized by immersing the slides in xylene. This was followed by rehydration through a series of graded ethanol solutions down to distilled water. After rehydration, the slides were treated with periodic acid solution for 5 min at room temperature. The slides were then thoroughly rinsed in distilled water four times to remove any residual periodic acid. Next, the sections were immersed in Schiff’s reagent for 15 min, during which the reagent reacted with the aldehyde groups to produce a magenta color, indicating the presence of glycogen in the tissue. After staining, the slides were rinsed again in distilled water to eliminate any excess Schiff’s reagent. Following the staining procedure, the slides were dehydrated through a graded ethanol series and cleared in xylene. The slides were then mounted and covered with a coverslip for microscopic examination.

The intensity of PAS staining, reflecting glycogen content in the muscle fibers, was quantitatively assessed using SigmaScan Pro 5 software (SPSS Inc., Chicago, IL, USA). Digital images of the stained tissue sections were captured under a light microscope, ensuring consistent magnification and lighting across all samples. For each tissue section, the software measured the intensity of staining in selected regions of interest, providing a grayscale value for each pixel. The intensity values ranged from 0, indicating no staining, to 255, representing the highest staining intensity. These measurements were used to determine the mean staining intensity for each sample, allowing for a quantitative comparison of glycogen levels between the control and experimental groups.

### Immunohistochemical (IHC) staining

To investigate the expression of specific proteins in the masseter muscle tissues, we conducted immunohistochemical staining using primary antibodies targeting Glut4 (catalog number sc-53566) from Santa Cruz Biotechnology (Santa Cruz, CA, USA), as well as p-AMPKα (catalog number 07–6814) from Merck (Rahway, NJ, USA). The tissue slides were initially prepared through a series of steps involving deparaffinization and hydration to remove the paraffin embedding medium and rehydrate the tissue sections. To prevent non-specific staining and enhance antigen retrieval, a peroxidase-blocking solution was applied to the slides. The slides were incubated with this blocking solution for 7 min at room temperature. After incubation, the slides were thoroughly rinsed with 1X phosphate-buffered saline (PBS) to remove excess blocking agent.

Subsequently, a protein block serum-free solution was applied to the slides to further reduce non-specific binding of antibodies. The slides were left undisturbed for one hour, allowing the blocking solution to occupy potential non-specific binding sites. For the detection of target proteins, the primary antibodies were diluted at a ratio of 1:100 in PBS. The diluted antibodies were added to the slides, ensuring complete coverage of the tissue sections. The slides were then incubated overnight at 4 °C to facilitate optimal binding between the antibodies and their specific antigens within the tissues.

The next day, the slides were given a thorough wash with 1X PBS to remove any unbound primary antibodies. Secondary antibodies, which are conjugated to an enzyme that reacts with a chromogenic substrate, were then applied to the slides. The slides were incubated with the secondary antibodies for 30 min at room temperature to allow binding to the primary antibodies. Following incubation with the secondary antibodies, the slides were washed again to remove unbound antibodies. A 3,3'-diaminobenzidine (DAB) solution was then applied to the slides for color development. DAB serves as a chromogen that produces a brown precipitate upon reaction with the enzyme linked to the secondary antibody, thereby visualizing the location of the target proteins within the tissue sections. For quantitative analysis, we utilized Sigma Scan Pro software to assess the extent of antibody staining. For each tissue section, the software measured the intensity of staining in selected regions of interest, providing a grayscale value for each pixel. The intensity values ranged from 0, indicating no staining, to 255, representing the highest staining intensity.

### Statistical analysis

All statistical analyses were performed using GraphPad Prism (version 10.3.1; GraphPad Software, Boston, MA, USA). The data were analyzed using a two-way analysis of variance (ANOVA) to evaluate the effects of two independent variables, diabetic status (diabetic vs. non-diabetic) and 4HR treatment (treated vs. untreated), on the dependent variable. Interaction effects between diabetic status and 4HR treatment were also assessed. Post-hoc pairwise comparisons were performed using Bonferroni’s multiple comparisons test to identify significant differences between specific groups. Data are presented as mean ± standard deviation. Statistical significance was defined as *p* < 0.05.

## Results

**Fig. 2:**
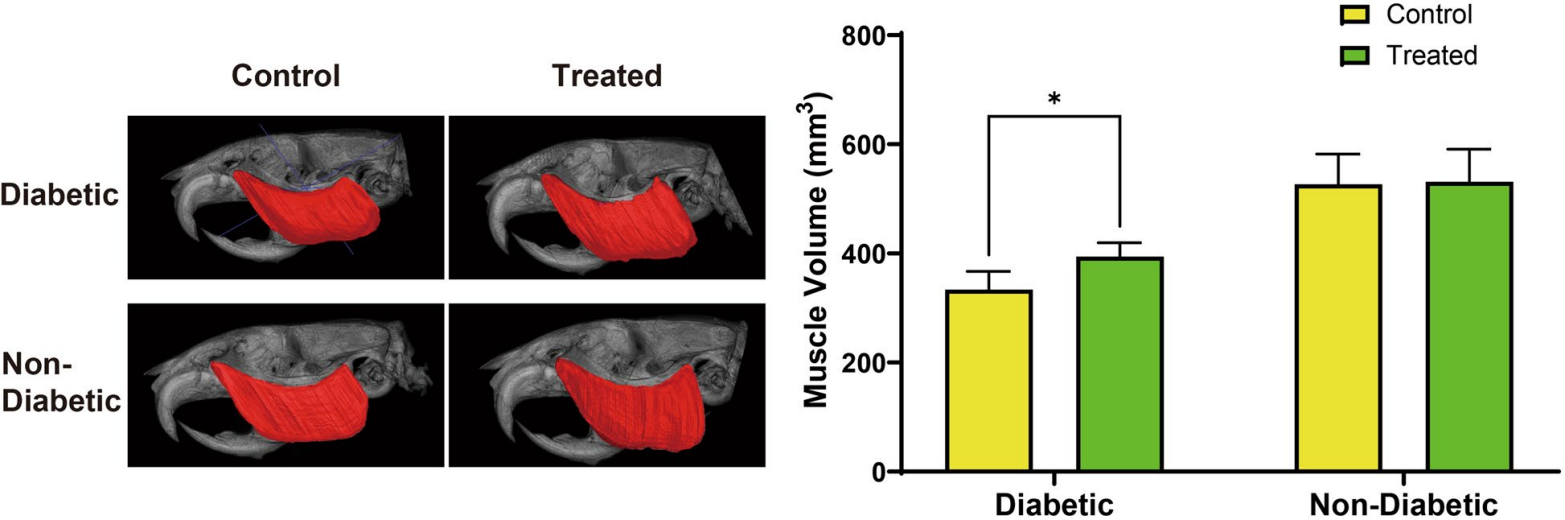
μCT analysis of masseter muscle volume in non-diabetic and diabetic rats with and without 4HR treatment. The masseter muscle was segmented and highlighted in red in the μCT images for volume measurement. μCT analysis demonstrated that the masseter muscle volume in diabetic groups was significantly smaller than in non-diabetic groups (^****^P < 0.001). Within the diabetic groups, the muscle volume was significantly smaller in the untreated group compared to the 4HR-treated group (.^*^P < 0.05)

Three-dimensional volume analysis using μCT demonstrated that the masseter muscle in diabetic groups was significantly smaller than in non-diabetic groups (Fig. 2). Two-way ANOVA revealed a highly significant main effect of diabetic status on muscle volume (F(1,34) = 119.04, *p* < 0.0001), with diabetes accounting for 74.08% of the total variance. A significant main effect of 4HR treatment was also observed (F(1,34) = 4.63, *p* = 0.0387), indicating that 4HR treatment influenced muscle volume, contributing to 2.879% of the total variance. The interaction between diabetic status and 4HR treatment was not statistically significant (F(1,34) = 3.43, *p* = 0.0726), though it accounted for 2.137% of the total variance. Post-hoc analysis indicated that 4HR treatment significantly increased muscle volume in diabetic animals (*p* < 0.05). These results suggest that 4HR treatment partially mitigates muscle atrophy associated with diabetes, though the effect is less pronounced in non-diabetic animals.

**Fig. 3:**
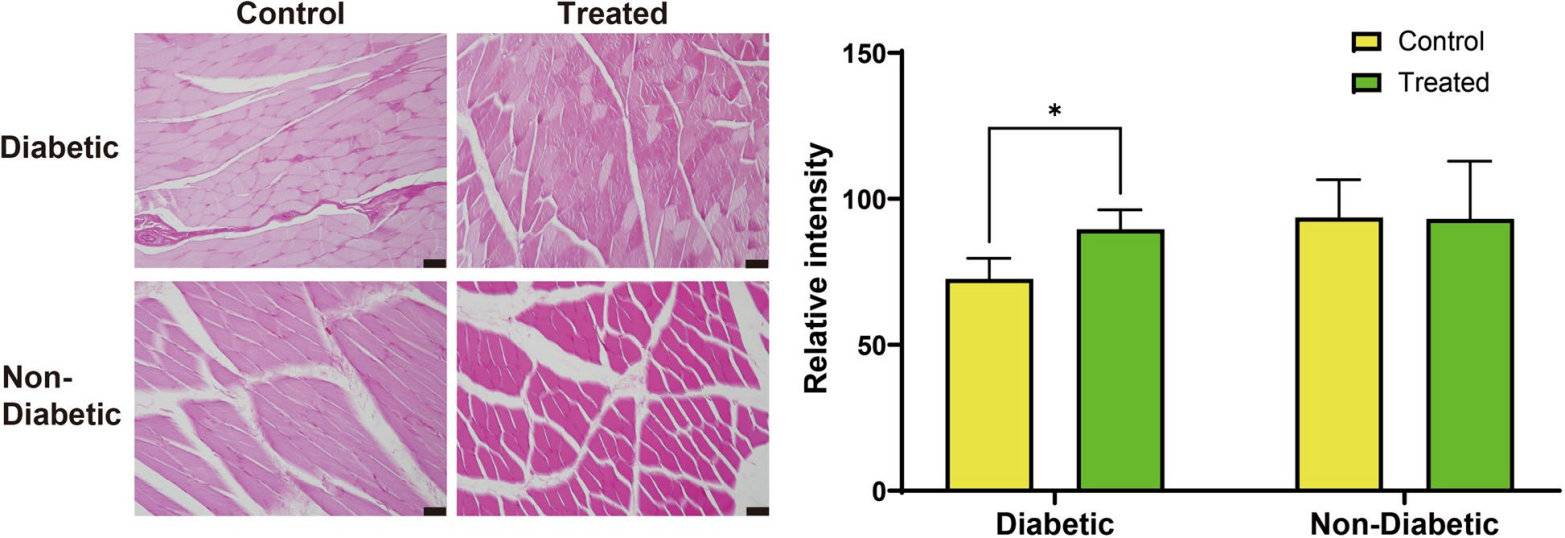
PAS-stained cross-sections of masseter muscle in diabetic rats with and without 4HR treatment. Photomicrographs of PAS-stained cross-sections from the masseter muscle of untreated diabetic rats and diabetic rats treated with 4HR are shown (scale bar = 50 μm; images without counterstaining). PAS staining intensity, which reflects glycogen content, was significantly lower in diabetic groups compared to non-diabetic groups (^**^P < 0.01). Within the diabetic groups, the PAS staining intensity was significantly lower in the untreated group than in the 4HR-treated group (.^*^P < 0.05), indicating that 4HR treatment mitigates the reduction in glycogen storage associated with diabetes (original magnification × 200)

Since oxidative muscle fibers appear lighter and glycolytic fibers appear darker with PAS staining, an increased ratio of glycolytic fibers results in higher PAS staining intensity (Fig. 3). Two-way ANOVA revealed a significant interaction between diabetic status and 4HR treatment on PAS intensity (F(1,34) = 4.24, *p* = 0.0472), accounting for 8.484% of the total variance. This indicates that the effect of 4HR treatment on PAS intensity differs depending on the diabetic status. There was a significant main effect of diabetic status (F(1,34) = 8.40, *p* = 0.0065), with diabetes reducing PAS intensity, accounting for 16.8% of the total variance. The main effect of 4HR treatment was not significant (F(1,34) = 3.76, *p* = 0.0609), suggesting that treatment alone did not substantially affect PAS intensity. Post-hoc analysis showed no significant difference in PAS intensity between the control group (93.61 ± 12.96) and the 4HR-treated group (93.10 ± 19.78) among non-diabetic animals. The diabetic groups exhibited lower PAS intensities compared to the non-diabetic groups. Within the diabetic groups, the mean PAS intensity was significantly lower in the untreated diabetic group (72.59 ± 7.03) compared to the 4HR-treated diabetic group (89.54 ± 6.73, *p* = 0.044). These findings suggest that diabetes reduces the ratio of glycolytic fibers, but 4HR treatment partially mitigates this effect, particularly in diabetic animals.

**Fig. 4:**
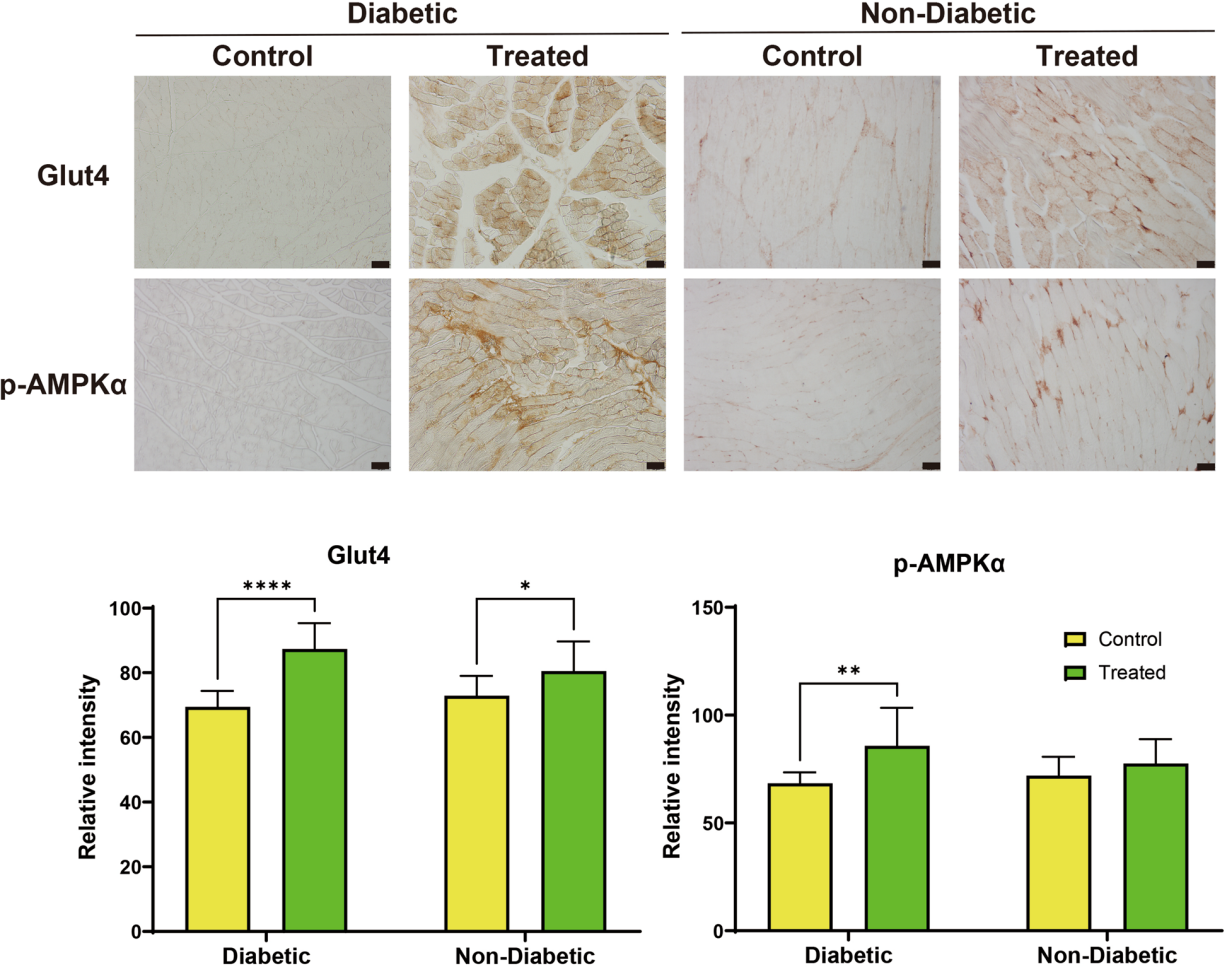
Immunohistochemical analysis of Glut4 and p-AMPKα expression in masseter muscle cross-sections from diabetic rats with and without 4HR treatment. Photomicrographs of masseter muscle cross-sections stained with anti-Glut4 and anti-p-AMPKα specific antibodies are shown (scale bar = 50 μm; images without counterstaining). The expression levels of Glut4 and p-AMPKα were significantly higher in the STZ/4HR group compared to the STZ group (^*^P < 0.05, ^**^.^**^P < 0.001), indicating that 4HR treatment enhances Glut4 and p-AMPKα expression in diabetic muscle tissue (original magnification × 200)

Immunohistochemistry analysis was performed to examine Glut4 and p-AMPKα staining intensities (Fig. 4). Two-way ANOVA revealed a significant interaction between diabetic status and 4HR treatment for Glut4 staining intensity (F(1,34) = 4.74, *p* = 0.0365), accounting for 7.046% of the total variance. This indicates that the effect of 4HR treatment on Glut4 expression differs depending on the diabetic status. The main effect of diabetic status was not significant (F(1,34) = 0.51, *p* = 0.4796), whereas the main effect of 4HR treatment was highly significant (F(1,34) = 29.18, *p* < 0.0001), explaining 43.36% of the variance. These findings suggest that 4HR treatment increases Glut4 expression, particularly in diabetic animals.

For p-AMPKα, two-way ANOVA showed no significant interaction between diabetic status and 4HR treatment (F(1,34) = 2.44, *p* = 0.1273), indicating that the effects of 4HR treatment on p-AMPKα expression do not depend on diabetic status. The main effect of diabetic status was not significant (F(1,34) = 0.38, *p* = 0.5436), while the main effect of 4HR treatment was very significant (F(1,34) = 9.42, *p* = 0.0042), accounting for 20.58% of the total variance. These results indicate that 4HR treatment significantly increases p-AMPKα levels regardless of diabetic status.

Post-hoc analysis showed that the 4HR-treated diabetic group had significantly higher Glut4 intensity (87.34 ± 7.97) compared to the untreated diabetic group (69.45 ± 4.93, *p* < 0.001), suggesting that 4HR enhances Glut4 expression under diabetic conditions. No significant difference was observed between the control (72.90 ± 6.13) and 4HR-treated (80.51 ± 9.15) groups (*p* > 0.05). For p-AMPKα, the 4HR-treated diabetic group (85.74 ± 17.65) exhibited significantly higher staining intensity compared to the untreated diabetic group (68.40 ± 5.06, *p* = 0.015), but there was no significant difference between the control (71.96 ± 8.68) and 4HR-treated groups (77.59 ± 11.25, *p* > 0.05). These findings suggest that 4HR treatment upregulates Glut4 and p-AMPKα expression, particularly in diabetic muscle tissue, potentially contributing to improved glucose uptake and metabolic function.

## Discussion

The U.K. Prospective Diabetes Study (UKPDS) and the Diabetes Control and Complications Trial (DCCT) have identified hyperglycemia as the primary cause of tissue damage in diabetes [23]. While genetic factors and individual susceptibility contribute to disease progression, persistently elevated blood glucose levels are considered the key initiating factor in the development of diabetic tissue damage. In a related study using the same animal model, 4HR administration significantly reduced blood glucose levels compared to the untreated diabetic group [20]. Furthermore, 4HR treatment increased the expression of Glut4 and p-AMPKα in hepatocytes, indicating improved glucose metabolism and cellular energy regulation [20]. Building on these findings, the present study aimed to evaluate the effects of 4HR on sarcopenia in the masseter muscle of an STZ-induced type I diabetic animal model and to elucidate the underlying molecular mechanisms. The results demonstrated that 4HR administration significantly alleviated sarcopenia in the masseter muscle (Fig. 2), likely through enhanced Glut4 expression and AMPK activation (Fig. 4).

First, μCT analysis revealed that 4HR administration alleviated sarcopenia in the masseter muscle (Fig. 2). Sarcopenia, characterized by the loss of muscle mass and strength, is a common complication associated with diabetes [24]. Poor glucose control and the presence of diabetic complications increase the risk of sarcopenia [4]. Reduced masticatory function is associated with diabetes mellitus [25], and better oral health, particularly masticatory function, is linked to a lower risk of sarcopenia and diabetes in older adults [26]. Our results suggest that 4HR has a protective effect against muscle wasting in this model, which is consistent with previous studies reporting the antioxidative and anti-inflammatory properties of 4HR [27, 28], known to mitigate muscle degeneration [29].

Second, histological analysis provided further evidence supporting the efficacy of 4HR. Increased PAS staining intensity, along with enhanced Glut4 and p-AMPKα immunostaining in the masseter muscle, were observed following 4HR treatment (Figs. 3 and 4). PAS staining is indicative of glycogen content in the tissue, and its increase suggests improved glucose storage and utilization in the muscle [30]. The immunostaining results corroborate the biochemical findings, highlighting the direct impact of 4HR on glucose metabolism and muscle energetics at the tissue level. Diabetes detrimentally affects muscle tissue through several mechanisms primarily driven by chronic hyperglycemia and metabolic disturbances [31]. Insulin resistance reduces glucose uptake in muscle cells, impairing energy metabolism and leading to muscle mass loss [32]. Concurrently, diabetes induces muscle atrophy via enhanced protein degradation pathways such as the ubiquitin–proteasome system (UPS) and autophagy [33]. Mitochondrial dysfunction results in decreased energy for muscle contraction, increasing fatigue and reducing strength [34]. Additionally, microvascular damage impairs nutrient and oxygen delivery to muscles, while diabetic neuropathy disrupts nerve signals essential for muscle coordination [35]. Chronic inflammation and oxidative stress further exacerbate muscle cell damage and degeneration, compounding the challenges in maintaining muscle health in diabetic individuals [36, 37].

One pivotal mechanism in diabetic myopathy is the role of endoplasmic reticulum (ER) stress in muscle degradation. In diabetic conditions, ER stress is markedly elevated, leading to muscle protein degradation through the UPS. Dysregulation of the UPS is a key contributor to muscle atrophy in diabetes, as increased levels of ubiquitin-activating enzyme and other UPS components enhance protein degradation, resulting in muscle atrophy and impaired muscle recovery [38]. Interventions that modulate ER stress and the UPS are crucial for preserving muscle mass and functionality in diabetes. Previous studies have shown that compounds like 4-phenylbutyric acid (4-PBA) effectively ameliorate muscle atrophy in diabetic rats through inhibition of ER stress and the UPS, reducing stress marker expression and mitigating protein degradation processes [39]. Similarly, Kim et al. [40] demonstrated that 4HR modulates ER and mitochondrial stress pathways in endothelial cells, increasing TGF-β1 expression. This suggests that 4HR may manage ER stress in muscle cells, potentially offering protective effects under conditions leading to atrophy. If 4HR can similarly modulate stress responses in muscle cells, it could help maintain muscle homeostasis and prevent degradation processes.

Moreover, 4HR’s antioxidative and anti-inflammatory properties may further contribute to its therapeutic benefits in diabetic myopathy. By suppressing TNF-α in host cells, 4HR could mitigate chronic inflammation that exacerbates muscle wasting in diabetes [41]. Its potential to reduce oxidative stress, either by scavenging free radicals or boosting antioxidant systems, could protect muscle cells from the damaging effects of diabetes, addressing both direct and indirect causes of muscle degradation [42]. Additionally, 4HR’s role in promoting angiogenesis could enhance muscle cell regeneration by improving blood flow and nutrient delivery, facilitating muscle repair and maintenance [43]. Collectively, these findings suggest that 4HR may offer therapeutic benefits in diabetic myopathy through multiple mechanisms, including modulation of ER stress, inhibition of protein degradation pathways, enhancement of glucose uptake via increased Glut4 expression and AMPK activation, and reduction of oxidative stress and inflammation. This presents a promising avenue for its application in managing diabetic myopathy, a condition characterized by muscle degeneration due to diabetes.

The rationale for selecting 4HR was firmly rooted in its diverse biological activities and potential therapeutic benefits in managing diabetes mellitus and its complications [44]. Song et al. [45] underscored 4HR as a novel inhibitor of α-glucosidase and non-enzymatic glycation, indicating its potential as a drug candidate for the prevention and treatment of type 2 diabetes mellitus. Our study contributes to this growing body of evidence by demonstrating that 4HR administration significantly alleviated sarcopenia in the masseter muscle of STZ-induced diabetic rats, likely through mechanisms involving enhancement of Glut4 expression and AMPK activation. Both 4-PBA and 4HR modulate stress responses within cells, particularly focusing on ER stress. Given 4HR’s demonstrated ability to increase TGF-β1 expression through ER and mitochondrial stress pathways in endothelial cells [40], it may similarly influence muscle cells, where ER stress and mitochondrial dysfunction are key contributors to atrophy. By potentially stabilizing cellular functions and preventing muscle protein degradation, 4HR could help in maintaining muscle homeostasis.

The current study has several limitations. First, while Glut4 expression and AMPK activation were assessed using immunohistochemistry, quantifying Glut4 levels and indices of AMPK activation, such as phospho-ACC levels, directly in the muscle tissue would provide more robust and precise data. Future studies should include such quantifications to strengthen the mechanistic understanding of 4HR’s effects on glucose metabolism and energy regulation. Second, this study focused on the masseter muscle due to its relevance in dentistry and its functional significance in mastication. However, skeletal muscles such as the gastrocnemius or soleus are more commonly studied in diabetic myopathy research, as they are more representative of systemic skeletal muscle changes. Future investigations should extend the evaluation of 4HR’s effects to these muscles to determine whether the findings observed in the masseter muscle are consistent across other skeletal muscles affected by diabetic sarcopenia. Addressing these limitations in future research would enhance the generalizability and mechanistic insights of 4HR’s therapeutic potential in combating diabetic myopathy.

## Conclusion

In conclusion, 4HR may offer therapeutic benefits in diabetic myopathy through several mechanisms. It can modulate ER stress, heavily implicated in the pathogenesis of the condition, and inhibit protein degradation pathways. Additionally, 4HR’s antioxidative and anti-inflammatory properties, along with its potential to promote angiogenesis, could further protect and regenerate muscle tissue. These results open new avenues for research into the effects of 4HR on skeletal muscle, particularly in the context of chronic conditions that precipitate muscle wasting.

## Consent for publication

Not applicable.

## Data Availability

No datasets were generated or analysed during the current study.

## Abbreviations

4HR: 4-Hexylresorcinol
4-PBA: 4-Phenylbutyric acid
ANOVA: One-way analysis of variance
BMPs: Bone morphogenetic proteins
DAB: 3,3'-Diaminobenzidine
DCCT: Diabetes Control and Complications Trial
ER: Endoplasmic reticulum
IHC: Immunohistochemical
NF-κB: Nuclear factor kappa B
p-AMPKα: Phosphorylated AMP-activated protein kinase alpha
PAS: Periodic acid–Schiff
STZ: Streptozotocin
TGF-β: Transforming growth factor-β
UKPDS: U.K. Prospective Diabetes Study
UPS: Ubiquitin–proteasome system
μCT: Micro-computed tomography

## References

[1] Cruz-Jentoft AJ, Bahat G, Bauer J, Boirie Y, Bruyère O, Cederholm T et al (2019) Sarcopenia: revised European consensus on definition and diagnosis. Age Ageing 48:16–31 30312372 10.1093/ageing/afy169 PMC6322506

[2] Mayhew A, Amog K, Phillips S, Parise G, McNicholas P, De Souza R et al (2019) The prevalence of sarcopenia in community-dwelling older adults, an exploration of differences between studies and within definitions: a systematic review and meta-analyses. Age Ageing 48:48–56 30052707 10.1093/ageing/afy106

[3] Qiao YS, Chai YH, Gong HJ, Zhuldyz Z, Stehouwer CD, Zhou JB et al (2021) The association between diabetes mellitus and risk of sarcopenia: accumulated evidences from observational studies. Front Endocrinol (Lausanne) 12:782391 35002965 10.3389/fendo.2021.782391 PMC8734040

[4] Feng L, Gao Q, Hu K, Wu M, Wang Z, Chen F et al (2022) Prevalence and risk factors of sarcopenia in patients with diabetes: a meta-analysis. J Clin Endocrinol Metab 107:1470–1483 34904651 10.1210/clinem/dgab884

[5] Hashimoto Y, Takahashi F, Okamura T, Hamaguchi M, Fukui M (2023) Diet, exercise, and pharmacotherapy for sarcopenia in people with diabetes. Metabolism 144:155585 37156410 10.1016/j.metabol.2023.155585

[6] Hirata Y, Nomura K, Senga Y, Okada Y, Kobayashi K, Okamoto S et al (2019) Hyperglycemia induces skeletal muscle atrophy via a WWP1/KLF15 axis. JCI Insight. 4:e124952 10.1172/jci.insight.124952 PMC6478420 30830866

[7] Schiaffino S, Mammucari C (2011) Regulation of skeletal muscle growth by the IGF1-Akt/PKB pathway: insights from genetic models. Skelet Muscle 1:1–14 21798082 10.1186/2044-5040-1-4 PMC3143906

[8] Tsalamandris S, Antonopoulos AS, Oikonomou E, Papamikroulis GA, Vogiatzi G, Papaioannou S et al (2019) The role of inflammation in diabetes: current concepts and future perspectives. Eur Cardiol Rev 14:50 10.15420/ecr.2018.33.1 PMC6523054 31131037

[9] Hao J, Wang Y, Huo L, Sun T, Zhen Y, Gao Z et al (2022) Circulating bone morphogenetic protein-9 is decreased in patients with type 2 diabetes and non-alcoholic fatty liver disease. Int J Gen Med 15:8539 36514745 10.2147/IJGM.S385513 PMC9741848

[10] Musi N, Goodyear LJ (2002) Targeting the AMP-activated protein kinase for the treatment of type 2 diabetes. Curr Drug Targets Immune Endocr Metabol Disord 2:119–127 12476786

[11] Joshi T, Singh AK, Haratipour P, Sah AN, Pandey AK, Naseri R et al (2019) Targeting AMPK signaling pathway by natural products for treatment of diabetes mellitus and its complications. J Cell Physiol 234:17212–17231 30916407 10.1002/jcp.28528

[12] Ha TKQ, Pham HTT, Cho HM, Tran VO, Yang JL, Jung DW et al (2019) 12,23-Dione dammarane triterpenes from Gynostemma longipes and their muscle cell proliferation activities via activation of the AMPK pathway. Sci Rep 9:1186 30718856 10.1038/s41598-018-37808-9 PMC6361897

[13] McGee SL, Van Denderen BJ, Howlett KF, Mollica J, Schertzer JD, Kemp BE et al (2008) AMP-activated protein kinase regulates GLUT4 transcription by phosphorylating histone deacetylase 5. Diabetes 57:860–867 18184930 10.2337/db07-0843

[14] Zavyalov O, Galimzhan D, Marina K (2022) Effect of feeding bioactive compounds identified from plant extracts (4-hexylresorcinol, 7-hydroxycoumarin, and gamma-octalactone) on the productivity and quality of broiler meat. Vet World 15:2986 36718328 10.14202/vetworld.2022.2986-2996 PMC9880825

[15] Song JY, Kim SG, Park Nr, Choi JY (2018) Porcine bone incorporated with 4-hexylresorcinol increases new bone formation by suppression of the nuclear factor kappa B signaling pathway. J Craniofac Surg. 29:1983–90 29561490 10.1097/SCS.0000000000004517

[16] Ahn J, Kim SG, Kim MK, Kim DW, Lee JH, Seok H et al (2016) Topical delivery of 4-hexylresorcinol promotes wound healing via tumor necrosis factor-α suppression. Burns 42:1534–1541 27198070 10.1016/j.burns.2016.04.016

[17] Kim DW, Jo YY, Garagiola U, Choi JY, Kang YJ, Oh JH et al (2020) Increased level of vascular endothelial growth factors by 4-hexylresorcinol is mediated by transforming growth factor-β1 and accelerates capillary regeneration in the burns in diabetic animals. Int J Mol Sci 21:3473 32423083 10.3390/ijms21103473 PMC7279008

[18] Lee JH, Kweon H, Oh JH, Kang YJ, Kim DW, Yang WG et al (2022) 4-Hexylresorcinol treatment before degumming increases the β-sheet structure of silk sericin and BMP-2 expression in RAW264.7. Int J Mol Sci. 24:150 36613594 10.3390/ijms24010150 PMC9820107

[19] Kim SG (2024) The Application of 4-Hexylresorcinol for Preventing Diabetic Complications. In: Kim SG (ed) Biomedical Application of 4-Hexylresorcinol. Springer, Berlin, pp 135–162

[20] Che X, Oh JH, Kang YJ, Kim DW, Kim SG, Choi JY et al (2024) 4-Hexylresorcinol enhances glut4 expression and glucose homeostasis via AMPK activation and histone H3 acetylation. Int J Mol Sci 25:12281 39596347 10.3390/ijms252212281 PMC11594624

[21] Lee IS, Kim DW, Oh JH, Lee SK, Choi JY, Kim SG et al (2021) Effects of 4-hexylresorcinol on craniofacial growth in rats. Int J Mol Sci 22:8935 34445640 10.3390/ijms22168935 PMC8396282

[22] Song KH, Kim MH, Jung JW, Kim AS, Hong SP, Kim SG (2009) The change in dimension of the masseter muscle in rabbits after radiofrequency therapy. J Oral Maxillofac Surg 67:485–490 19231770 10.1016/j.joms.2008.06.051

[23] Brownlee M (2005) The pathobiology of diabetic complications: a unifying mechanism. Diabetes 54:1615–1625 15919781 10.2337/diabetes.54.6.1615

[24] Lisco G, Disoteo OE, De Tullio A, De Geronimo V, Giagulli VA, Monzani F et al (2023) Sarcopenia and diabetes: A detrimental liaison of advancing age. Nutrients 16:63 38201893 10.3390/nu16010063 PMC10780932

[25] Yamazaki T, Yamori M, Asai K, Nakano-Araki I, Yamaguchi A, Takahashi K et al (2013) Mastication and risk for diabetes in a Japanese population: a cross-sectional study. PLoS One. 8:e64113 10.1371/journal.pone.0064113 PMC3674007 23755114

[26] Abe T, Tominaga K, Ando Y, Toyama Y, Takeda M, Yamasaki M et al (2021) Number of teeth and masticatory function are associated with sarcopenia and diabetes mellitus status among community-dwelling older adults: A Shimane CoHRE study. PLoS One. 16:e0252625 10.1371/journal.pone.0252625 PMC8172058 34077486

[27] Yen GC, Duh PD, Lin CW (2003) Effects of resveratrol and 4-hexylresorcinol on hydrogen peroxide-induced oxidative DNA damage in human lymphocytes. Free Radic Res 37:509–514 12797471 10.1080/1071576031000083099

[28] Liu J, Chen B, Hu Q, Zhang Q, Huang B, Fei P (2023) Pectin grafted with resorcinol and 4-hexylresorcinol: preparation, characterization and application in meat preservation. Int J Biol Macromol 237:124212 36977442 10.1016/j.ijbiomac.2023.124212

[29] Zhao W, Li Y, Cheng X, Wei H, Li P, Fan L et al (2023) The antioxidant Glycitin protects against intervertebral disc degeneration through antagonizing inflammation and oxidative stress in nucleus pulposus cells. Aging 15:13693 38019477 10.18632/aging.205251 PMC10756108

[30] Zou F, Mao XQ, Wang N, Liu J, Ou-Yang JP (2009) Astragalus polysaccharides alleviates glucose toxicity and restores glucose homeostasis in diabetic states via activation of AMPK. Acta Pharmacol Sin 30:1607–1615 19960007 10.1038/aps.2009.168 PMC4007496

[31] Sran S, Sran M, Ferguson N, Anand P (2014) Diabetic myonecrosis: uncommon complications in common diseases. Case Rep Endocrinol 2014:175029 24716004 10.1155/2014/175029 PMC3970048

[32] Abdul-Ghani MA, DeFronzo RA (2010) Pathogenesis of insulin resistance in skeletal muscle. J Biomed Biotechnol 2010:476279 20445742 10.1155/2010/476279 PMC2860140

[33] Shen Y, Li M, Wang K, Qi G, Liu H, Wang W et al (2022) Diabetic Muscular Atrophy: Molecular Mechanisms and Promising Therapies. Front Endocrinol 13:917113 10.3389/fendo.2022.917113 PMC9279556 35846289

[34] Yamada T, Ivarsson N, Hernández A, Fahlström A, Cheng AJ, Zhang SJ et al (2012) Impaired mitochondrial respiration and decreased fatigue resistance followed by severe muscle weakness in skeletal muscle of mitochondrial DNA mutator mice. J Physiol 590:6187–6197 22988144 10.1113/jphysiol.2012.240077 PMC3530125

[35] Flynn MD, Tooke JE (1995) Diabetic neuropathy and the microcirculation. Diabet Med 12:298–301 7600742 10.1111/j.1464-5491.1995.tb00480.x

[36] D’Souza DM, Al-Sajee D, Hawke TJ (2013) Diabetic myopathy: impact of diabetes mellitus on skeletal muscle progenitor cells. Front Physiol 4:379 24391596 10.3389/fphys.2013.00379 PMC3868943

[37] Hernández-Ochoa EO, Vanegas C (2015) Diabetic myopathy and mechanisms of disease. Biochem Pharmacol Open Access. 4:e179 10.4172/2167-0501.1000e179 PMC4675628 26693099

[38] Reddy SS, Shruthi K, Prabhakar YK, Sailaja G, Reddy GB (2018) Implication of altered ubiquitin-proteasome system and ER stress in the muscle atrophy of diabetic rats. Arch Biochem Biophys 639:16–25 29277369 10.1016/j.abb.2017.12.015

[39] Reddy SS, Shruthi K, Joy D, Reddy GB (2019) 4-PBA prevents diabetic muscle atrophy in rats by modulating ER stress response and ubiquitin-proteasome system. Chem Biol Interact 306:70–77 30980806 10.1016/j.cbi.2019.04.009

[40] Kim JY, Kim DW, Lee SK, Choi JY, Che X, Kim SG et al (2021) Increased expression of TGF-β1 by 4-hexylresorcinol is mediated by endoplasmic reticulum and mitochondrial stress in human umbilical endothelial vein cells. Appl Sci 11:9128

[41] Kim SG (2022) 4-Hexylresorcinol: Pharmacologic chaperone and its application for wound healing. Maxillofac Plast Reconstr Surg 44:5 35103875 10.1186/s40902-022-00334-w PMC8805429

[42] Chang JH, Kim DW, Kim SG, Kim TW (2021) Alleviation of oxidative stress in dental pulp cells following 4-hexylresorcinol administration in a rat model. Appl Sci 11:3637

[43] Kim MK, Kim SG, Lee SK (2020) 4-Hexylresorcinol induced angiogenesis potential in human endothelial cells. Maxillofac Plast Reconstr Surg 42:1–11 32642459 10.1186/s40902-020-00267-2 PMC7324454

[44] Jeong H, Kim JY, Che X, Choi JY, Jang I, Kim SG (2023) Effects of 4-hexylresorcinol on facial skeletal development in growing rats: Considerations for diabetes. Korean J Orthod 53:393 37989576 10.4041/kjod23.091 PMC10663577

[45] Song S, Liu Q, Chai WM, Xia SS, Yu ZY, Wei QM (2021) Inhibitory potential of 4-hexylresorcinol against α-glucosidase and non-enzymatic glycation: Activity and mechanism. J Biosci Bioeng 131:241–249 33191127 10.1016/j.jbiosc.2020.10.011

